# A Randomized, Double-Blind Pilot Trial of Hydrolyzed Rice Bran versus Placebo for Radioprotective Effect on Acute Gastroenteritis Secondary to Chemoradiotherapy in Patients with Cervical Cancer

**DOI:** 10.1155/2015/974390

**Published:** 2015-11-26

**Authors:** Yoshiyuki Itoh, Mika Mizuno, Mitsuru Ikeda, Rie Nakahara, Seiji Kubota, Junji Ito, Tohru Okada, Mariko Kawamura, Fumitaka Kikkawa, Shinji Naganawa

**Affiliations:** ^1^Department of Radiology, Nagoya University Graduate School of Medicine, Nagoya, Aichi 4668550, Japan; ^2^Department of Obstetrics and Gynecology, Nagoya University Graduate School of Medicine, Nagoya, Aichi 4668550, Japan; ^3^Department of Radiological Technology, Nagoya University Graduate School of Medicine, Nagoya, Aichi 4610047, Japan

## Abstract

We aimed to evaluate the radioprotective effect of hydrolyzed rice bran (HRB) on acute gastroenteritis due to chemoradiotherapy for treatment of cervical cancer. This placebo-controlled, double-blind study was conducted as an exploratory investigation of the colitis-inhibiting effects of HRB in alleviating acute-phase gastrointestinal side effects of chemoradiotherapy. The study involved 20 patients (10 in the HRB group, 10 in the control group). The patients in the control group underwent the same chemoradiotherapy regimen as those in the HRB group, but they received a placebo instead of HRB. The diarrheal side effect assessment score was lower in the HRB than control group, and a trend toward a reduction in diarrhea symptoms was observed with the oral intake of HRB. Additionally, no significant difference was observed in the administration of intestinal regulators and antidiarrheal agents, but again the assessment score was lower in the HRB than control group, and diarrhea symptoms were alleviated with the oral intake of HRB. A trend toward no need for strong antidiarrheal agents was seen. Although this study was an exploratory clinical trial, the results suggest that HRB may relieve diarrhea, an acute-phase gastrointestinal side effect of chemoradiotherapy.

## 1. Introduction

Radical treatment for cervical cancer generally involves surgical treatment or radiation therapy, either alone or in combination. Recent reports have described better treatment outcomes with chemoradiotherapy than with radiation therapy alone [1–4]. Standard radiation therapy for cervical cancer includes brachytherapy with or after external-beam radiation therapy (EBRT). Because a large part of the pelvic region is irradiated during EBRT, much of the healthy tissue of the small intestine, colon, and rectum is included in the radiation field. The severity of side effects (adverse events) in the acute phase depends on the volume of the irradiation field [5]. Gastrointestinal side effects of radiotherapy in the acute phase include abdominal pain and diarrhea secondary to radiation enteritis. Acute-phase mucosal symptoms involving the urinary system include frequent urination and blood in the urine secondary to radiation cystitis and urethritis. All of these adverse effects decrease patients' quality of life during treatment. When chemotherapy is combined with radiation for added potency, these acute-phase symptoms increase in severity [6, 7]. Intensity-modulated radiation therapy, a new radiotherapy technique that attempts to reduce the dose of radiation to normal tissue, is one means of reducing gastrointestinal symptoms [5, 7].

In current clinical practice, diarrhea accompanying radiation therapy is symptomatically treated by administration of intestinal regulators, antidiarrheal agents, and similar medications [8, 9]. Therefore, an effective therapy to alleviate the inflammatory effects that occur with radiation therapy is needed. Hydrolyzed rice bran (HRB) (Daiwa Pharmaceutical Co., Ltd., Tokyo, Japan) is a processed food, the basic ingredient of which is water-soluble rice bran fiber. Trials of its effectiveness and safety have been conducted in animals and humans. HRB exhibited a significant inflammation-inhibitory effect in an experimental murine model of dextran sulfate-induced colitis when compared with the control group (unpublished data). Another study found that HRB may have an anti-inflammatory effect by inhibition of mast cell degranulation and cytokine production in bone marrow-derived mast cells [10]. Furthermore, an investigation of the effects of HRB on the prevalence of the common cold syndrome and severity of cold symptoms in older patients was performed [11]. Although no significant difference was seen in the prevalence of the cold syndrome between the HRB and control groups, the severity of symptoms was significantly milder in the HRB than control group.

Therefore, to verify the colitis-inhibiting action of HRB, we conducted an exploratory investigation of the ability of HRB to alleviate acute-phase gastrointestinal side effects of chemoradiotherapy in patients with cervical cancer. In animals HRB enhances NK activity of aged mice and may be useful for enhancing NK function in humans [12, 13]. We also evaluated the changes in natural killer (NK) cell activity as an immune-enhancing action with the combined use of HRB.

## 2. Patients and Methods

### 2.1. Patients

All patients in this trial underwent chemoradiotherapy for cervical cancer. Patients with primary squamous cell carcinoma, adenocarcinoma, or adenosquamous carcinoma located in the cervix were included. Patients with small cell carcinoma or sarcoma were excluded. The present study was approved by the investigational review board of our hospital (number 993), and written informed consent was obtained from all patients included in this study. This clinical trial is registered in the UMIN Clinical Trials Registry (UMIN000004350).

The inclusion criteria were as follows: (i) age of ≥20 to <75 years at the time of providing informed consent; (ii) cervical cancer with the intent for chemoradiotherapy; (iii) adequately maintained major organ function (bone marrow, liver, and kidneys) and laboratory parameters within the following ranges: white blood cell count of >3500/mm^3^, absolute neutrophil count of >1500/mm^3^, hemoglobin A1c level of ≥10.0 g/dL, platelet count of ≥100,000/mm^3^, total bilirubin level of ≤1.5 mg/dL, aspartate transaminase and alanine transaminase levels of <80 IU/L, serum creatinine level of <1.5 mg/dL, and creatinine clearance rate of ≥60 mL/min (Cockcroft-Gault formula or 24 h creatinine clearance); and (iv) having received an explanation of the purpose and methods of this trial and having provided written consent prior to the start of the trial.

The exclusion criteria were as follows: (i) undergoing surgical treatment; (ii) undergoing a nonsurgical treatment thought to affect treatment with HRB and its outcome; (iii) presence of a drug allergy; (iv) known or possible pregnancy, desire to become pregnant, or currently breastfeeding; and (v) other conditions that the principal investigator or a coresearcher thought they might make an individual unsuitable for this study.

The study sample comprised 20 patients (10 in the HRB group, 10 in the control group). This sample size was based on the fact that this was an exploratory study and did not have enough statistical power. This was a pilot study to calculate the efficacy rate of HRB, which is necessary to estimate sample sizes for later studies, and to collect safety information.

### 2.2. Methods

This was a single-institution, randomized, placebo-controlled, double-blind study conducted at Nagoya University Hospital. The HRB tested in this trial was a processed food, the main component of which was a partial hydrolysate of rice bran produced by the action of polysaccharide hydrolytic enzymes of the supernatant of a mycelium culture of* Lentinula edodes* in water-soluble rice bran fiber. The main component of the placebo food substance given to the control group was dextrin, a water-soluble polysaccharide, and it did not include HRB. Both food substances were given in the form of granules sealed on three sides in an aluminum foil processed film of the same shape and were indistinguishable by appearance. This study coordinator encodes two food substances with matching random numbers, and patients are randomly assigned to the control or HRB group. Neither patient, nor their doctor knows until the end of this trial. Patients who gave written informed consent were randomly assigned to either the HRB group or the control group.

In the HRB group, three packets of the HRB (1 g of HRB per packet) were taken orally three times per day. In the control group, three packets of the placebo food were taken orally three times per day. The HRB or placebo was consumed before the start of chemoradiotherapy (up to 1 week before) and it was taken every day during receiving radiation therapy. Its use of each drug has been also stopped simultaneously with EBRT end.

The survey measures were patient characteristics, clinical laboratory test parameters, and gastrointestinal symptoms. The patient characteristics included diagnosis, birthdate, age, sex, body weight, inpatient/outpatient status, medical history, and complications. The clinical laboratory tests included blood tests (erythrocyte count, hemoglobin level, hematocrit, white blood cell count, and platelet count), serum chemistry tests (levels of aspartate transaminase, alanine transaminase, gamma-glutamyl transpeptidase, alkaline phosphatase, total bilirubin, total protein, total cholesterol, lactate dehydrogenase, triglycerides, uric acid, blood urea nitrogen, and creatinine), and NK cell activity. The gastrointestinal symptoms evaluated were diarrhea, nausea, vomiting, and lack of appetite; both their frequency and severity were assessed.

The time course of the study is shown in Table 1.

The primary endpoint of this study was the frequency and severity of diarrhea symptoms, and the secondary endpoints were the frequency and severity of gastrointestinal symptoms other than diarrhea (nausea, vomiting, and loss of appetite) and NK cell activity. NK cell activity was compared between the two study groups before intake of the HRB or placebo and at the completion of radiation therapy. All symptoms were scored numerically according to the following criteria.

*Diarrhea.* 0, no symptoms; 1, mild symptoms (bowel movements up to 3 times more frequent than normal; passage of stool slightly more frequent than normal); 2, moderate symptoms (bowel 4 to 6 times more frequent than normal; <24-hour intravenous drip is ordered; passage of stool moderately more frequent than normal, but this does not interfere with daily life); and 3, severe symptoms (bowel movements ≥7 times more frequent than normal; incontinence; ≥24-hour intravenous drip is ordered, hospitalization necessary; passage of stool is markedly more frequent than normal and interferes with daily life). 

*Administration of Intestinal Regulators and Antidiarrheal Agents.* 0, not administered; 1, administration of agents containing lactic acid bacilli or bifidobacteria (*Lactobacillus casei* or* Clostridium butyricum*); 2, administration of agents containing albumin tannate or berberine chloride; and 3, administration of agents containing loperamide hydrochloride. 

*Nausea.* 0, no symptoms; 1, mild symptoms (loss of appetite without change in eating habits); 2, moderate symptoms (decreased oral intake without marked loss of weight, dehydration symptoms, or undernourishment; <24-hour intravenous drip is ordered); and 3, severe symptoms (poor intake of calories and water by mouth; ≥24-hour intravenous drip, enteral feeding, or total parenteral nutrition is ordered). 

*Vomiting.* 0, no symptoms; 1, mild symptoms (once in 24 hours); 2, moderate symptoms (2–5 times in 24 hours; <24-hour intravenous drip is ordered); and 3, severe symptoms (≥6 times in 24 hours; ≥24-hour intravenous drip or total parenteral nutrition is ordered). 

*Loss of Appetite.* 0, no symptoms; 1, mild symptoms (loss of appetite without change in eating habits); 2, moderate symptoms (decreased oral intake without marked loss of weight or undernourishment; oral nutrition supplements ordered); and 3, severe symptoms (decreased oral intake with marked loss of weight or undernourishment; drip infusion, enteral feeding, or total parenteral nutrition is ordered).

### 2.3. Safety Assessment

Safety assessment was performed by evaluating laboratory values before and upon completion of taking the HRB, and they were graded by the National Cancer Institute scale (Common Terminology Criteria for Adverse Events, v3.0 [12]). Also, grade refers to the severity of the diarrhea according to the CTCAE v3.0, but when there was no adverse event, it was made grade “0.” Grade 4 is the adverse event associated with life-threatening consequences and it was excluded from this grading.

### 2.4. Treatment for Cervical Cancer

Radiation therapy involved a combination of EBRT and brachytherapy. EBRT was performed at 1.8 Gy once per day (total dose of 50.4 Gy in 28 fractions). Central shielding was performed upon starting brachytherapy. EBRT was applied at 1.8 Gy once a day, five times a week (Monday–Friday) (total dose of 50.4 Gy in 28 fractions). When whole-pelvis radiation was performed, four-field radiation of the anterior-posterior and two lateral portals was used as a rule. The radiotherapy methods used in our institute are detailed elsewhere [13]. EBRT alone was performed in patients who could not undergo brachytherapy for some reason (e.g., the tandem and ovoid instruments could not be inserted because of a markedly narrow vagina; deformation was present due pubic bone or other pelvic fractures, or instruments could not be inserted for some other reason).

The following chemotherapy regimen was performed every 3 weeks: cisplatin at 70 mg/m^2^ on day 1 and a continuous infusion of 5-fluorouracil at 700 mg/m^2^ on days 1 to 4.

### 2.5. Statistical Analysis

The differences in the side effect assessment scores between the HRB and control groups were compared with Fisher's exact test. The NK cell activity values and white blood cell counts were analyzed by repeated-measures analysis of variance. A *P* value of <0.05 was taken to indicate a statistically significant difference.

## 3. Results

### 3.1. Study Population

**Figure 1:**
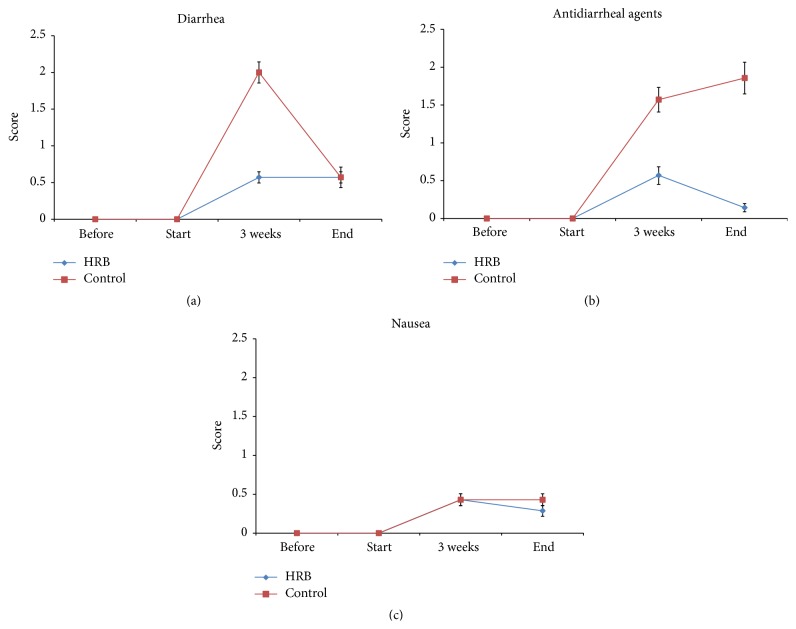
(a) Effects of HRB on diarrhea after chemoradiotherapy. (b) Effects of HRB on use of antidiarrheal agents after chemoradiotherapy. (c) Effects of HRB on nausea after chemoradiotherapy.

The patients' median age was 47.5 years (range, 30–72 years). The histological types of cervical cancer were squamous cell carcinoma in 18 patients and adenosquamous carcinoma in 2. In all 20 patients with cervical cancer, treatment began with chemoradiotherapy, but, in 2 of the 20 patients, the radiation therapy was discontinued about 1 month after it was started, and switched to surgery. These two patients were therefore excluded from the assessments. We also excluded another four patients in whom the ingestion rate of the test food was low (ingestion rate of 0% at both 3 weeks and at completion; all of these patients had strong nausea and vomiting due to the chemoradiotherapy and consumed almost nothing by mouth). After excluding these 6 patients, we assessed the results of 14 patients (HRB group, 7 patients; control group, 7 patients). The frequency and severity of diarrhea, the primary endpoint, were not significantly different between the HRB and control groups in the third week of radiation therapy, but the diarrheal side effect assessment score was lower in the HRB group, and a trend was seen toward a reduction in diarrhea symptoms with oral intake of HRB group (Figure 1(a)). There was also no significant difference in the administration of intestinal regulators and antidiarrheal agents, but again the assessment score was lower in the HRB group than in the control group, and diarrhea symptoms were alleviated with oral intake of HRB. A trend was observed toward no need for strong antidiarrheal agents (Figure 1(b)). No significant differences in nausea (Figure 1(c)), vomiting, or loss of appetite were seen between the HRB and control groups.

**Figure 2:**
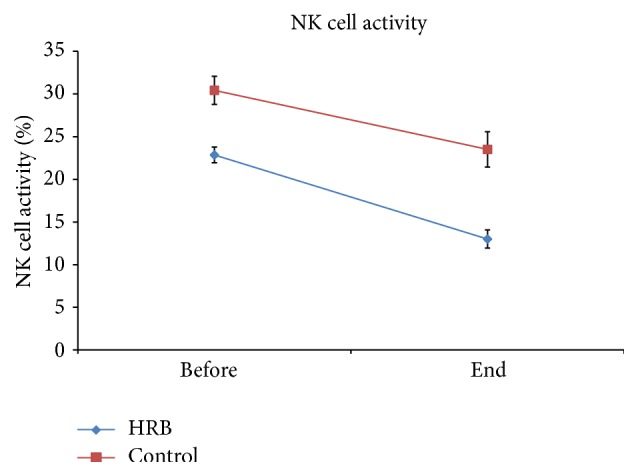
NK cell activity before and after chemoradiotherapy.

Similarly, no large difference in NK cell activity was seen between the two groups (Figure 2).

### 3.2. Safety Assessment

Blood test and serum chemistry results were compared between before and at the end of consumption of the test food. The white blood cell counts before and after the treatment are shown in Table 2. Patients with high values were seen in both the HRB and control groups, but in all of these patients, the high counts were judged to be an inflammatory response to the cancer (most were pyometra). The white blood cell counts were also significantly lower in both groups at the end of chemoradiotherapy, but this was considered to be mainly a side effect of the chemoradiotherapy.

There was no significant interaction between the two groups in the decrease of the white blood cell count. However, five of seven patients in the HRB group showed normal white blood cell counts, whereas three of seven patients in the control group showed normal white blood cell counts. When expressed by National Cancer Institute grading, two patients in the HRB group developed grade 2 adverse events, while one, two, and one patient in the control group developed grade 1, 2, and 3 adverse events, respectively. The control group tended to develop adverse events of higher grades than did the HRB group. Furthermore, when the grade was the same, the white blood cell counts tended to be lower in the control group (Table 2).

No marked differences were seen in the other items between the HRB and control groups. Observed side effects (adverse events) were hypokalemia in one patient in the HRB group and cystitis in one patient in the control group. In both cases, however, the physicians diagnosed the side effects as unrelated to consumption of the test food.

## 4. Discussion

HRB is a processed food, the basic ingredient of which is water-soluble rice bran fiber. In this study, we paid attention to the colitis-inhibiting action of HRB and conducted an exploratory investigation of its effect in relieving acute-phase gastrointestinal side effects of chemoradiotherapy for cervical cancer. We set the frequency and severity of diarrhea symptoms as the primary endpoint, and the frequency and severity of gastrointestinal symptoms other than diarrhea (nausea, vomiting, and loss of appetite) as the secondary endpoint. However, because these items are affected by patients' subjective symptoms, we performed a prospective, randomized, placebo-controlled, double-blind study for a more rigorous assessment of the effects of HRB. However, the sample size did not have enough statistical power because this study was an exploratory study to determine the efficacy of the test food, serve as a baseline to determine the sample size of future detailed studies, and gather information.

In this study, no significant difference was detected in the primary and secondary endpoints between the two groups. However, because diarrhea, a side effect of chemoradiotherapy, in the HRB group showed an inclination to be less frequent than that in the control group, oral intake of HRB may enable a reduction in the dose of drugs generally administered to treat diarrhea and other gastrointestinal side effects in the acute phase [8, 9]. This could make it possible to continue treatment while maintaining patients' quality of life during the treatment. There was no significant difference in the decrease of NK cell activity before and after intake of the test food between the HRB and control groups. However, decreases in the white blood cell count were seen in only two of seven patients in the HRB group, whereas such decreases were seen in four of seven patients in the control group. With respect to adverse events, only two patients developed grade 2 adverse events in the HRB group, while one, two, and one patient in the control group developed grade 1, 2, and 3 adverse events, respectively. Thus, more severe side effects occurred in the control group. Furthermore, when both groups exhibited the same grade 2 adverse events, the white blood cell count itself tended to be lower in the control group (Table 2).

This study was an exploratory clinical trial conducted as a pilot study with a small number of patients. However, the results suggest that HRB may relieve diarrhea, an acute-phase gastrointestinal side effect of chemoradiotherapy for cervical cancer, and may inhibit the decrease in white blood cells that occurs with chemoradiotherapy. Additionally, no safety problems were observed in this study. While there were no significant differences between the two groups, the results seem promising with respect to the efficacy of HRB in reducing diarrhea symptoms and inhibiting a decrease in the white blood cell count. A multicenter, large-scale prospective clinical trial will be needed to confirm these benefits of HRB intake.

## 5. Conclusion

Our study suggested that HRB may relieve diarrhea, an acute-phase gastrointestinal side effect of chemoradiotherapy in patients with cervical cancer, although this randomized, double-blind study is exploratory and also a sample size is very small.

## Figures and Tables

**Table 1:** Time course of the study.

Items	Before uptake	Treatment				Stop of this study
		Before	Start	3 weeks	End	
Informed consent	◯					
Subjective or objective complaints	◯		◯	◯	◯	◯
Clinical laboratory tests	◯				◯	◯
NK cell activity	◯				◯	
Test food (HRB or placebo food)		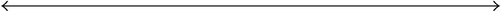				
Chemoradiotherapy		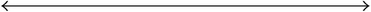				
Adverse events		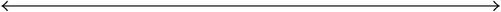				

**Table 2:** White blood cell counts before and after treatment.

	Age	WBC (cells/*μ*L)		Grade (after)
		Before	After	
HRB	64	8700	2500	2
	40	10000	3700	0
	70	7700	3400	0
	62	10300	5600	0
	44	7000	4000	0
	31	8900	2600	2
	38	6400	5700	0

Control	72	6300	3200	1
	58	8200	3400	0
	69	10200	4600	0
	51	10200	2000	2
	52	7900	1600	3
	40	11000	2400	2
	57	6600	3400	0
